# Nanoengineered mitochondria for mitochondrial dysfunction and anti-aging interventions

**DOI:** 10.3389/fragi.2025.1688482

**Published:** 2025-09-26

**Authors:** Siqi Deng, Yingying Ren, Qian Zhang, Qinling Liu, Jiaxin Long, Kelsey Picard, Miguel Martin, Thomas Miller, Chaofan Yuan, Yunxiang He, Junling Guo

**Affiliations:** ^1^ BMI Center for Biomass Materials and Nanointerfaces, National Engineering Laboratory for Clean Technology of Leather Manufacture, Key Laboratory of Leather Chemistry and Engineering (Sichuan University), Ministry of Education, College of Biomass Science and Engineering, Sichuan University, Chengdu, Sichuan, China; ^2^ Tea Refining and Innovation Key Laboratory of Sichuan Province, College of Horticulture, Sichuan Agricultural University, Chengdu, Sichuan, China; ^3^ Division of 64K Cellssense, Vitagenix Asia Research & Innovation, Hong Kong SAR, China; ^4^ State Key Laboratory of Polymer Materials Engineering, Sichuan University, Chengdu, Sichuan, China; ^5^ Department of Chemical and Biological Engineering, Bioproducts Institute, The University of British Columbia, Vancouver, BC, Canada

**Keywords:** anti-aging, nanoengineered mitochondrial biohybrids, surface functionalization, mitochondrial function restoration, age-related diseases

## Abstract

Aging is a multifactorial process and a major risk factor for chronic disease. Among its hallmarks, mitochondrial dysfunction plays a central role, driven by impaired respiration and accumulated mitochondrial DNA mutations that disrupt energy metabolism and redox balance. Conventional mitochondrial transplantation has been explored as a therapeutic strategy, but its emphasis on increasing mitochondrial quantity without restoring function has limited success. Recent advances in nanoengineered mitochondria that integrate isolated mitochondria with functional nanomaterials, offer new opportunities to enhance organelle quality, boost metabolic activity, and achieve targeted delivery. Preclinical studies highlight their promise in cardiovascular, neurodegenerative, and other age-related disorders. In this mini-review, mitochondrial dysfunction in aging is first introduced, followed by the summary of rational designed strategies for engineering mitochondrial biohybrids and their emerging applications, and finally translational challenges are further discussed. By bridging materials science and mitochondrial therapy, nanoengineered mitochondria may represent a next-generation approach to anti-aging interventions.

## 1 Introduction

Aging represents one of the most pressing global challenges in the coming decades (Li et al., 2024). According to the World Health Organization, the population aged 60 years and older is projected to double from 1 billion in 2020 to 2.1 billion by 2050, highlighting the immense scale of global aging. The development of age-related pathologies not only seriously impacts the health of the elderly population but also imposes a socioeconomic burden, driving the urgent need for effective anti-aging strategies (Tuttle et al., 2021). A growing body of research has revealed that aging is driven by the accumulation of diverse cellular insults, including mitochondrial dysfunction, genomic instability, and oxidative stress. Specifically, mitochondria are essential regulators of energy metabolism, signaling, and cell fate (Guo et al., 2023; Suomalainen and Nunnari, 2024), but their function declines with age, marked by impaired oxidative phosphorylation (OXPHOS), accumulation of mitochondrial deoxyribonucleic acid (mtDNA) mutations, dysregulation of the tricarboxylic acid (TCA) cycle, and elevated levels of reactive oxygen species (ROS) (Sun et al., 2016). This functional decline is not merely a measurement of aging but actively drives the aging, positioning mitochondria as both triggers and amplifiers of cellular senescence.

Naturally derived mitochondrial modulators emerge as prominent anti-aging strategies, with mainstream antioxidant components including calcium α-ketoglutarate (AKG) (Ames, 2018; Zhang et al., 2023), ergothioneine (EGT) (Chin et al., 2014; Zhao et al., 2024c), ubiquinone-10 (Díaz-Casado et al., 2019), selenium (Bjørklund et al., 2022; Huang et al., 2024), and resveratrol (Lagouge et al., 2006; Drago et al., 2024). AKG, a key intermediate in the TCA cycle, and EGT, a thiol-histidine derivative, act synergistically to support mitochondrial homeostasis and systemic resilience against age-associated stress. However, their delivery efficiency and targeting precision remain largely underdeveloped. Meanwhile, conventional mitochondrial transplantation (MT), a therapeutic process involving the isolation and delivery of healthy exogenous mitochondria to damaged cells or organs to restore bioenergetics and promote repair, typically relies on the direct injection or infusion of isolated, unmodified mitochondria (Headley and Tsao, 2023). MT has been shown to restore cardiomyocyte and neuronal function across various disease contexts. Preclinical studies further demonstrate that MT can revive adenosine triphosphate (ATP) production, alleviate oxidative stress, and improve organ function in models of ischemia and neurodegeneration (Sun et al., 2020; Mukherjee et al., 2021). Although conventional MT increases mitochondrial quantity, this unmodified delivery approach fails to enhance the quality and activity of individual mitochondria, thereby limiting its therapeutic potential against aging and related pathologies (Riou et al., 2025).

**FIGURE 1:**
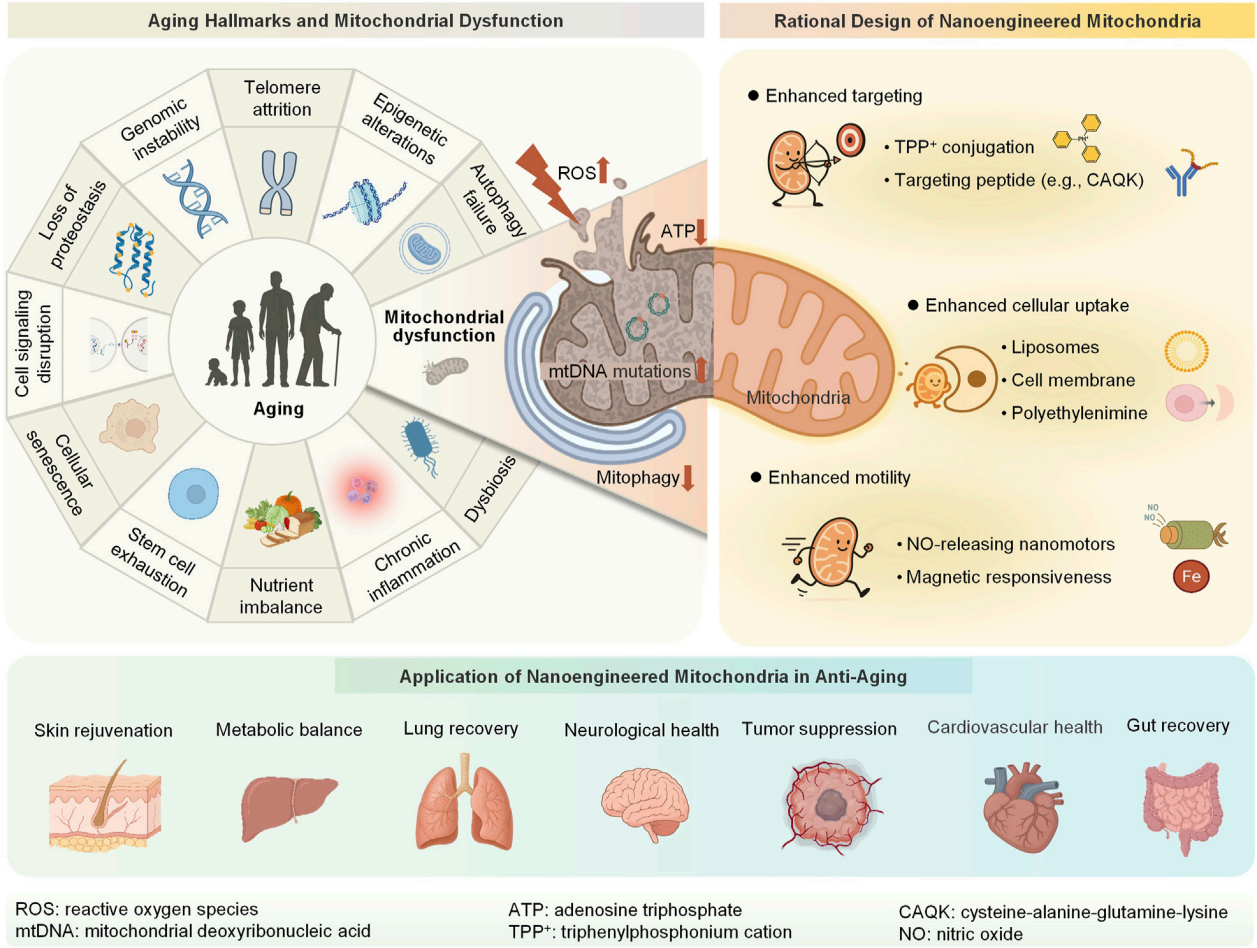
Schematic overview of the relationship between aging and mitochondrial dysfunction, the design of nanoengineered mitochondria, and their applications in anti-aging therapy. ROS, reactive oxygen species; mtDNA, mitochondrial deoxyribonucleic acid; ATP, adenosine triphosphate; TPP^+^, triphenylphosphonium cation; NO, nitric oxide; CAQK, cysteine-alanine-glutamine-lysine (Created with BioRender.com).

Inspired by cell surface engineering, we propose nanoengineered mitochondria, which are biohybrid systems formed by integrating synthetic nanomaterials or biomolecules with isolated mitochondria to confer new functionalities (He et al., 2024; He et al., 2025; Liu et al., 2025). This emerging strategy operates at the interface of bioengineering and mitochondrial biology and aims to overcome the limitations of conventional MT (Guo et al., 2014; Qiu et al., 2021). These tailored nanobiohybrid systems have the potential to improve mitochondrial quality, boost metabolic activity, and reduce ROS levels (Chen et al., 2025; Wang et al., 2025a; Wang et al., 2025c). Moreover, these systems can enhance the targeting efficiency and motility of mitochondria, which is achieved through mitochondrial ligand-receptor recognition (e.g., triphenylphosphonium cation (TPP^+^)-modified nanoparticles and mitochondrial membrane potentials) (Zeng et al., 2025), stimulus-responsive navigation (e.g., pH/ROS-sensitive polymers guiding mitochondria to inflammatory sites), and external field-driven propulsion (e.g., magnetically steered nanocapsules). This mini-review therefore focuses specifically on the emerging of nanoengineered mitochondria, moving beyond the scope of earlier reviews that centered primarily on conventional transplantation. We first discuss the central role of mitochondrial dysfunction in aging and present recent advances in the rational design of nanoengineered mitochondria. We then highlight their therapeutic potential in treating age-related diseases and conclude by critically evaluating translational challenges and future directions (Figure 1). Collectively, we envision nanoengineered mitochondria as a next-generation platform for precise anti-aging interventions.

## 2 Mechanisms of mitochondrial dysfunction in aging

Aging is a complex and multifactorial biological process marked by the gradual and irreversible loss of cellular homeostasis and regenerative capacity, serving as a major risk factor for chronic diseases such as neurodegeneration, cardiovascular dysfunction, metabolic disorders, and cancer (Guo et al., 2022). In 2023, López-Otín et al. proposed twelve hallmarks of aging (López-Otín et al., 2023). Among these, mitochondrial dysfunction represents a major driver of aging, arising through a multilayered chain of causes and effects (Somasundaram et al., 2024). The accumulation of mtDNA mutations reduces electron transport chain efficiency and elevates ROS production, and a commonly proposed hypothesis is that ROS-induced damage further amplifies mitochondrial dysfunction, forming a self-reinforcing cycle (Nandi et al., 2019; Houldsworth, 2024). However, it remains unclear whether this cycle represents a primary driver of systemic aging or merely constitutes a secondary consequence of other pathological processes, a fundamental question that remains a major research gap in the field. In parallel, reduced activity of the PTEN-induced putative kinase 1 (PINK1)–PARKIN RBR E3 ubiquitin ligase (PARKIN) pathway impairs mitophagy, allowing defective mitochondria to persist and thereby exacerbating neurodegeneration and inflammation (Pickrell and Youle, 2015; Fang et al., 2019; Picca et al., 2023; Narendra and Youle, 2024). Furthermore, declines in respiratory chain function and ATP supply particularly compromise homeostasis in high-energy-demand tissues such as the brain and heart (Desler et al., 2012; Gasmi et al., 2024). Notably, the relative contribution of bioenergetic deficit versus oxidative damage to aging phenotypes across different tissues is still not fully quantified. Disruption of the AMP-activated protein kinase (AMPK)–Sirtuin 1 (SIRT1)–Peroxisome proliferator-activated receptor gamma coactivator 1-alpha (PGC-1α) signaling axis further impedes mitochondrial biogenesis and metabolic flexibility, promoting inflammation and senescence (Toyama et al., 2016; Zhu et al., 2025).

Collectively, these mitochondrial impairments converge to accelerate aging, underscoring the urgent need for integrated strategies to restore mitochondrial quality and function. By contrast, nutrient supplements such as AKG and EGT mitigate dysfunction through multiple mechanisms. AKG fuels energy metabolism, scavenges ROS, protects membranes, and supports Fe^2+^/AKG-dependent dioxygenases, thereby driving epigenetic remodeling and delaying senescence. It also activates the AMPK–SIRT1–PGC-1α axis, promoting mitochondrial biogenesis (Cheng et al., 2024). EGT accumulates in mitochondria, scavenges ROS, preserves mtDNA integrity, and maintains cristae structure while enhancing respiration through sulfur-transferase activity and redox cycling (Chin et al., 2014; Ames, 2018).

## 3 Rational design of nanoengineered mitochondria

While nutritional supplements such as AKG and EGT offer a promising, multi-mechanistic approach to ameliorate mitochondrial dysfunction, their efficacy remains constrained by poor bioavailability, insufficient tissue-specific targeting, and the complexity of age-related damage. To overcome these challenges, nanoengineered mitochondria have emerged as a promising alternative. Nanoengineered mitochondria are biohybrid systems in which isolated mitochondria are integrated with functional nanomaterials, including inorganic particles, organic polymers, and biomacromolecules. This integration confers improved targeting, enhanced motility, and greater cellular internalization. In practice, mitochondrial modification is commonly achieved through small peptide labeling, liposome transfection, vesicle packaging, or polymer coating (Zhao et al., 2024). These approaches provide versatile platforms for tailoring mitochondrial functions and can be categorized into strategies that enhance targeting, motility, and cellular internalization, with representative examples summarized in Table 1.

**TABLE 1:** Summary of rational design strategies for nanoengineered mitochondria.

Strategy	Functions	Design	Mechanism	Model	Advantages	Limitations
Liposome	Enhancing internalization (Nakano et al., 2022)	Coating mitochondria with DOTAP/DOPE lipid bilayers	Cationic lipid coating enhances surface charge and stability, improving uptake and lowering immunogenicity	Neurons *in vitro*; mouse cerebral ischemia model	Improved surface charge and structural stability; higher uptake; improved neuroprotection	Risk of immune activation; potential toxicity of cationic lipids
	Enhancing internalization (Hu et al., 2024)	Fusion of mitochondria with lung-targeted liposomes containing ROS scavenger (Tempol)	Liposome fusion enables lung targeting and loads scavenger to reduce ROS and inflammation	Macrophages *in vitro*; ARDS mouse model	Enhanced lung accumulation; reduced cytokines and ROS; preserved mitochondrial activity	Manufacturing complexity; circulation half-life constraints
	Enhancing targeting (Sivagnanam et al., 2023)	Liposomes functionalized with TPP and dansyl fluorophore	TPP attraction assists drug delivery to mitochondria	Cancer cell lines (HCT116, MCF7)	Improved targeting of chemotherapeutics; inducing apoptosis	Limited tissue penetration; *in vitro* only; limited systemic data
Chemical conjugation	Enhancing targeting (Sun et al., 2023)	Conjugation of PEP peptide and TPP to mitochondria	TPP attraction assists delivery and PEP peptide enables targeting to ischemic myocardium	Mouse ischemia-reperfusion model	Non-invasive delivery; efficient targeting to ischemic heart	Peptide stability in circulation; limited to acute injury models
	Enhancing targeting (Xu et al., 2024)	Conjugation of CAQK peptide and TPP to mitochondria	CAQK peptide binds proteoglycans at SCI lesions; TPP enhances uptake	Mouse spinal cord injury model	Specific accumulation at lesion; improved macrophage phagocytosis; tissue regeneration	Safety concerns in chronic SCI; CAQK peptide/TPP toxicity
Surface functionalization	Enhancing internalization (Zhang et al., 2024)	Coating of mannosylated PEI to mitochondria (mPEI/M1mt)	Mannose-functionalized, mPEI-coated M1-mitochondria preferentially internalize into M2-TAMs and reprogram to M1	Tumor-associated macrophages (TAMs); mouse tumor models	Higher uptake efficiency; selective internalization; enhances checkpoint therapy	Potential polymer toxicity, limited *in vivo* stability
	Enhancing targeting (Zhang et al., 2024)	Decoration of mitochondria with ROS/Ca^2+^-responsive hydrogel and engineered EVs loading PINK1 mRNA	Engineered EVs stimulates mitochondrial autophagy and the hydrogel maintains mitochondrial mass equilibrium	Peripheral nerve injury	Responsive release; enhanced regeneration	Delivery complexity; Hybrid approach; hydrogel stability uncertain
	Enhancing motility (Wu et al., 2024)	Nanomotorized mitochondria with surface coating of NO-releasing polymer and CM coating	Nanomotor enables chemotaxis toward iNOS/ROS, while CM fragments promote uptake and accumulation	Oral delivery in IHD animal model	Active navigation, non-invasive oral administration; chemotaxis enhances targeting; high retention	Limited oral bioavailability; Complex design; scalability concerns
	Enhancing motility (Li et al., 2025)	Construction of artificial mitochondrial nanorobots (PFMACr AMNs) with polymeric nanoparticles containing PCr shuttle	MAPCr drives propulsion and PFA enables barrier penetration, allowing PFMACr AMNs to sustain mitochondrial motilityin hypoxia	Hypoxia-injured H9c2 cells; myocardial infarction mouse model	Self-propulsion, barrier penetration; sustained mitochondrial motility under hypoxia	Fully synthetic; may not integrate with endogenous mitochondria

TPP^+^, triphenylphosphonium cation; CAQK, cysteine-alanine-glutamine-lysine peptide; PEP, ischemia-targeting peptide (CSTSMLKAC); EV, extracellular vesicle; PINK1, PTEN-induced putative kinase 1; NO, nitric oxide; CM, cardiomyocyte membrane; AMNs, artificial mitochondrial nanorobots; MAPCr, N-methacryloyl-L-arginine-phosphocreatine; PFA, perfluorooctyl acrylate; LMR, lung-mitochondria ROS, scavenger; DOTAP, 1,2-dioleoyl-3-trimethylammonium propane; DOPE, L-α-dioleoyl phosphatidylethanolamine; TAMs, tumor-associated macrophages; ROS, reactive oxygen species; iNOS, inducible nitric oxide synthase; ARDS, acute respiratory distress syndrome; IHD, ischemic heart disease; SCI, spinal cord injury; TME, tumor microenvironment; ATP, adenosine triphosphate; mtDNA, mitochondrial DNA.

### 3.1 Enhancing mitochondrial targeting function

Liposomes functionalized with mitochondrial-targeting moieties represent a prominent platform for targeted drug delivery and mitochondrial modulation (Gajbhiye et al., 2023). TPP^+^ possesses a cationic and lipophilic nature and can accumulate in mitochondria while maintaining low reactivity and ease of synthesis (Kulkarni et al., 2021; Kaneko et al., 2023; Pawar et al., 2023). The conjugation of TPP^+^ enables specific targeting to cancer cell mitochondria, facilitating the delivery of drugs to trigger apoptosis (Reshetnikov et al., 2018). For instance, liposomes of TPP^+^-functionalized 10,12-pentacosadiynoic acid in phospholipids provide a stable cationic ligand with fluorescent labeling, enabling both targeting and visualization of mitochondrial interactions (Sivagnanam et al., 2023). To further improve targeting specificity, TPP^+^ was conjugated with cysteine-alanine-glutamine-lysine (CAQK) peptides (Xu et al., 2024). This design facilitated strong mitochondrial binding by inserting into the outer mitochondrial membrane to form the Mito-TPP-CAQK compound, thereby improving delivery precision. Building on the previous development as a versatile linker, TPP^+^ was used to connect the ischemia-targeting peptide (PEP, CSTSMLKAC) to mitochondria (Sun et al., 2023), to generate a PEP-TPP-mitochondria compound, where an enhanced cellular internalization into AC16 cardiomyocytes was observed compared with unmodified mitochondria, highlighting the potential of functionalized liposomes for cardiac applications. Beyond synthetic liposomes, adaptive hydrogel systems have also been explored to construct nanoengineered mitochondria to regulate the functions. In a model of Wallerian degeneration (WD) post-peripheral nerve injury, adaptive hydrogels have been used to deliver engineered extracellular vesicles (E-EV-P@HPCEP) carrying PINK1 mRNA (Zhao et al., 2024a). These EVs target senescent Schwann cells, stimulate mitochondrial autophagy, and maintain mitochondrial mass balance, thereby mitigating WD progression and improving nerve repair outcomes. Taken together, these findings suggest that liposomes equipped with mitochondrial-targeting ligands, especially TPP^+^-based constructs, offer a versatile and efficient strategy for mitochondrial drug delivery.

### 3.2 Enhancing mitochondrial motility

Nanoengineered mitochondria offer a promising approach to enhance mitochondrial motility, which played a significant role in the therapeutic outcomes in cardiac tissues (Sun et al., 2025). Mitochondria modified with nitric oxide (NO)-releasing nanomotors, to generate nanomotorized mitochondria (NM/Mito), can be guided toward cardiac lesions via chemotactic migration, characterized by high levels of inducible nitric oxide synthase (iNOS) and ROS (Wu et al., 2024). Further coating the cardiomyocyte membrane (CM) fragments asymmetrically to the surface of NM/Mito to generate CM/NM/Mito and further loading into pH-responsive enteric capsules to generate CM/NM/Mito@Cap, a non-invasive mitochondrial transplant strategy was developed via oral administration. CM/NM/Mito showed time-dependent fluorescence enhancement in high ROS/iNOS reservoirs, indicating active chemotaxis along the concentration gradient. Encapsulated in enteric-coated capsules for oral delivery, the formulation avoids gastric acid degradation and targets myocardial tissues, aiding ischemic heart disease (IHD) treatment. In parallel, monomers with motility (N-methacryloyl-L-arginine-phosphocreatine, MAPCr) and trans-barrier (Perfluorooctyl acrylate, PFA) units were employed to synthesize artificial mitochondrial nanorobots (PFMACr AMNs) (Li et al., 2025). The design of PFMACr AMNs enables the provision of high-energy phosphate bonds to damaged mitochondria, and enhances motility within pathological microenvironments.

### 3.3 Enhancing mitochondrial internalization

Surface functionalization with targeting ligands and bioactive molecules have been widely explored to enhance mitochondrial internalization, thereby improving the therapeutic efficacy of transplanted mitochondria in tissue repair (Wang et al., 2024a). A representative example is the development of a lung-targeted, mitochondria-based ROS scavenging system, termed LMR (lung-mitochondria ROS scavenger). LMR was fabricated by fusing mitochondria-targeting liposomes with lung-targeting liposomes encapsulating the ROS scavenger Tempol (Hu et al., 2024). The liposomes were prepared from dimethyldioctadecylammonium bromide, cholesterol, and 1,2-distearoyl-sn-glycero-3-phosphoethanolamine–polyethylene glycol 2000-Tempol (DSPE-PEG2000-Tempol) using a method of solvent evaporation. In an ARDS model, LMR showed enhanced lung accumulation, preserved mitochondrial activity, and alleviated inflammation compared with unmodified mitochondria. An alternative strategy to enhance mitochondrial delivery is coating isolated mitochondria with a cationic artificial lipid membrane. Using a modified inverted emulsion method, mitochondria were encapsulated with 1,2-dioleoyl-3-trimethylammonium propane/L-α-dioleoyl phosphatidyl ethanolamine (DOTAP/DOPE) lipids, generating artificial membrane-coated mitochondria (AM-mito) with improved surface charge and structural stability. This coating not only preserved key mitochondrial proteins and membrane potential but also significantly enhanced cellular internalization and neuroprotective efficacy *in vitro* and *in vivo* (Nakano et al., 2022). Compared to unmodified mitochondria, AM-mito demonstrated uptake by neurons under ischemic conditions and reduced brain infarct volume following cerebral ischemia-reperfusion injury. By further exploring mitochondrial surface engineering, low-molecular-weight polyethyleneimine (mPEI) was used to coat isolated mitochondria from M1 macrophages, resulting in mannose-functionalized mitochondria (mPEI/M1mt) (Zhang et al., 2024). This modification enhanced mitochondrial uptake by tumor-associated macrophages (TAMs) and promoted their phenotypic shift toward pro-inflammatory M1 macrophages.

## 4 Application of nanoengineered mitochondria in anti-aging

Building on these rational design strategies, nanoengineered mitochondria have been applied in diverse preclinical models to rejuvenate mitochondrial performance and restore cellular homeostasis. Integrated with functional nanomaterials, these systems extend the therapeutic scope of conventional mitochondrial transplantation and have shown promise across a wide range of pathologies, including cancer, acute respiratory distress syndrome (ARDS), spinal cord injury, and peripheral nerve damage (Hu et al., 2024; Wang et al., 2025b; Yang et al., 2025). In the context of aging, their applications can be broadly categorized into three strategies: activation of mitochondrial autophagy, nanoengineered approaches in mitochondrial transplantation, and enhancement of ATP production capacity.

### 4.1 Activation of mitochondrial autophagy

Reduction of mitochondrial autophagy contributes to aging and is generally associated with the accumulation of damaged mitochondria, further linked to various age-related diseases such as pulmonary fibrosis, erectile dysfunction, and cardiovascular diseases. A recent study developed mitophagy-enhanced nanoengineered mitochondria (Mito-MEN) by anchoring PARKIN mRNA-loaded nanoparticles to healthy mitochondria, which not only improved exogenous mitochondrial delivery but also activated mitophagy to eliminate endogenous damaged mitochondria (Wang et al., 2024b). In a pulmonary fibrosis model, Mito-MEN restored mitochondrial pool homeostasis and significantly alleviated fibrosis symptoms, demonstrating the potential of nanoengineered mitochondria in treating aging-related lung disease. A schematic design of a piezoelectric nanosystem was developed for the treatment of diabetes-related erectile dysfunction (Wang et al., 2025b). In an erectile dysfunction model, the piezoelectric co-loaded nanosystem induced current that promoted mitochondrial autophagy and reduced ROS generation, thereby decreasing blood glucose levels and protecting mitochondria from damage. Each component of this nanosystem functions independently or cooperatively, thereby promoting penile repair and restoring erectile function.

### 4.2 Improvement of mitochondrial transplantation

MT technology, by delivering healthy mitochondria to damaged cells, tissues, or organs, has emerged as a potential therapeutic approach for treating mtDNA-related diseases and restoring the mitochondrial function of diseased cells in recent years (Zhao et al., 2024b). For example, PARKIN mRNA-loaded nanoparticle-engineered mitochondria (mNP-Mito) enhanced the delivery efficiency of healthy exogenous mitochondria and the mitophagy of damaged mitochondria, thereby restoring the function of complex I in treating Leber hereditary optic neuropathy (Wang et al., 2024c). Moreover, surface-engineered mitochondria that reprogram TAMs from pro-tumor M2 to anti-tumor M1 phenotype (mPEI/M1mt) through immunometabolic modulation can significantly enhance checkpoint inhibitor therapy in murine cancer models (Zhang et al., 2024). The transplantation of mPEI/M1mt reshapes the tumor immune microenvironment by promoting the activation and infiltration of CD8^+^ and CD4^+^ T cells. Specifically, CD8^+^ T cells act as the principal cytotoxic effectors, releasing perforin and granzymes to directly induce apoptosis of tumor cells (Koh et al., 2023). In parallel, CD4^+^ T helper cells orchestrate immune responses by secreting cytokines such as Interleukin-2 (IL-2) and Interferon-gamma (IFN-γ), which sustain CD8^+^ T cell proliferation and function, while also supporting dendritic cell maturation and antigen presentation (Topchyan et al., 2023). The coordinated action of cytotoxic and helper T cells is further strengthened by surface-engineered mitochondrial transplantation, which restores bioenergetics and promotes immune reprogramming, thereby synergizing with anti-Programmed Death-Ligand 1 (PD-L1) treatment to achieve superior antitumor efficacy.

### 4.3 Enhancement of mitochondrial ATP production

Damage to the ATP-producing capacity of mitochondria directly affects the energy supply of cells, leading to a series of diseases. Mitochondria-based nanorobots (PFMACr AMNs) can offer sufficient energy by manipulating the internal phosphate bond to effectively treat ischemic heart disease (Li et al., 2025). By co-incubating PFMACr AMNs with hypoxia-injured H9c2 cells, it was found that PFMACr AMNs were able to synthesize ATP for 12 h, keeping the ATP level in hypoxia-injured H9c2 cells comparable to that in normal H9c2 cells. In a myocardial infarction mouse model, PFMACr AMNs sustained ATP levels and reduced infarct size, demonstrating superior therapeutic potential. This innovative design opens up a new path for the construction of an artificial energy delivery system in the body. Despite these advances, most applications of nanoengineered mitochondria remain limited to rodent models, with large-animal and early human data still lacking. This gap represents a major barrier to clinical translation.

## 5 Conclusion

Functional nanomaterials, ranging from versatile inorganic and organic materials to biomacromolecules, have been integrated with mitochondria to construct engineered systems endowed with enhanced targeting, motility, and internalization efficiency. In addition to increasing mitochondrial biogenesis, this strategy also strengthens mitochondrial respiration and energy production. We reviewed mitochondrial dysfunction in aging, outlined design strategies of nanoengineered mitochondria, and summarized recent advances in their applications to age-related diseases. Collectively, these advances pave the way for the development of next-generation subcellular therapies targeting aging and its related disorders. Nevertheless, the clinical translation of nanoengineered mitochondria is still at an early stage, and several major challenges remain.

### 5.1 Delivery efficiency and stability

Mitochondria are fragile organelles, and preserving their structural and functional integrity during systemic administration remains technically challenging. Oral delivery, while advantageous for patient compliance, still requires improved tissue penetration, biological stability, and bioavailability. Potential strategies include polymeric or lipid coatings, encapsulation within hydrogels or microcapsules, and vesicle-based delivery systems to protect mitochondria and improve biodistribution. In addition, artificial intelligence-assisted design may help optimize material-mitochondria interactions and guide the development of more efficient delivery formulations.

### 5.2 Safety and immunogenicity

Long-term biosafety and immunogenicity need careful evaluation in both autologous and allogeneic contexts. While surface functionalization improves targeting efficiency, it may also cause off-target accumulation, immune responses, or interference with host metabolism. Future research should therefore emphasize systematic biosafety assessment and the development of low-immunogenic coatings or immune-evasive surface modifications to ensure safe translation.

### 5.3 Mechanistic understanding and therapeutic enhancement

The molecular mechanisms by which nanoengineered mitochondria interact with host signaling pathways remain incompletely understood. It is critical to clarify their roles in mitochondrial biogenesis, mitophagy, the AMPK–SIRT1–PGC-1α axis, and immunometabolic reprogramming. In parallel, incorporating widely studied anti-aging components such as AKG, EGT, selenium, ubiquinone-10, and resveratrol into nanoengineered mitochondrial systems may enhance mitochondrial quality, improve redox homeostasis, and expand therapeutic potential. Advanced tools such as organoid-based disease models, gene editing, and systems-level analyses can further accelerate mechanistic insights and support rational design.
